# Employing drug delivery strategies to create safe and effective pharmaceuticals for COVID‐19

**DOI:** 10.1002/btm2.10163

**Published:** 2020-05-13

**Authors:** Kevin J. McHugh

**Affiliations:** ^1^ Department of Bioengineering Rice University Houston TX

The outbreak of the novel SARS‐CoV‐2 pathogen and corresponding coronavirus disease 2019 (COVID‐19) have had an enormous impact on both global health and the daily lives of billions of people worldwide. With a proven vaccine at least a year from being fully tested for safety and efficacy, there may be an opportunity to rapidly repurpose existing drugs in order to prevent SARS‐CoV‐2 infections and improve outcomes for patients already infected with COVID‐19. At present, more than 40 different drugs are being explored for efficacy against COVID‐19, including antivirals and immune modulating compounds. Unfortunately, many of these drugs are associated with side effects that limit their use to the most severe cases and thereby prevent their use as prophylactics. This commentary describes drug formulation strategies that can be used to maintain the efficacy of these drugs through controlled release, targeted delivery, and nonviral nucleic acid delivery. If successful, these approaches could enable the expanded use of drugs to reduce the mortality of this devastating disease and equip healthcare providers with the tools to accelerate our recovery from this pandemic and improve our response to the next outbreak of a novel pathogenic virus.

The paradigm that prevention is more effective than treatment holds true across much of medicine. Vaccination against infectious disease, which is responsible for some of the greatest and most cost‐effective improvements in public health, is perhaps the best example of this principle in action.^1^ Despite confidence expressed by the United States and other countries prior to SARS‐CoV‐2, it has become clear that we were ill‐prepared to rapidly respond and mitigate a viral outbreak with a comprehensive response plan. Put simply, we have failed to provide our citizens with the tools necessary to stop the spread of coronavirus disease 2019 (COVID‐19). This failure is most glaring in the lack of protection for healthcare workers, who have lacked adequate access to the personal protective equipment (PPE) they rely on to avoid contracting the disease themselves.^2^ We have, in short order, met “Disease X,” the unexpected and severe infectious disease that the World Health Organization and others such as Bill Gates had feared could quickly escalate and become an worldwide pandemic.^3^, ^4^ Thus far, a majority of COVID‐19's impact has been felt in countries that are most integrated into the global economy and are fairly well equipped from a healthcare perspective. If (or more likely when) the disease reaches critical levels in low‐ and middle‐income countries, we expect to see an increase in the death rate from our current estimate of ~1%^5^, ^6^ due to the lack of adequate medical facilities and equipment as well as quarantining procedures that are more difficult to implement in those settings.^7^

In response to the global COVID‐19 pandemic, there has been a major emphasis on developing a vaccine for SARS‐CoV‐2—and rightfully so. Herd immunity for COVID‐19 does not appear likely to come to our rescue,^8^ so developing a vaccine that confers long‐lived protection is, and should be, our primary goal. However, our ability to develop a vaccine suitable for clinical use in a timely manner remains to be seen. Even with new strategies in vaccine development, which enabled Moderna (Cambridge, MA) and the National Institute of Allergy and Infectious Disease (NIAID) to design, produce, and administer their mRNA‐1,273 vaccine to humans in Phase I clinical trials just 63 days after the viral genome sequence was first reported,^9^ there is still a long way to go before it, or another vaccine, is proven to be safe and effective. While the FDA can and seems willing to reduce the regulatory burdens that can otherwise slow down progress, there is an immunological limit to the speed at which clinical trials can be ethically performed.^10^ This point has been underscored by previous reports from similar coronaviruses showing that anti‐spike IgG antibodies induced by an experimental vaccine was complicit in promoting a pro‐inflammatory reaction in the lung, exacerbating acute respiratory distress syndrome (ARDS), and potentially leading to death.^11^, ^12^ Once approved, it must then be manufactured at scale, though the parallel creation of vaccine production facilities customized for each of the top candidate vaccines currently underway could speed this process.^13^ Therefore, while a vaccine may ultimately be our savior, current best‐case scenario estimates put the availability of a clinically viable vaccine at 12–18 months.^14^, ^15^ Even that would be a two‐ or threefold improvement compared to the original mumps vaccine, which holds the record for the shortest time between virus isolation and vaccine development (1945–1948). Unfortunately, that vaccine yielded only short‐term protection and was replaced several decades later by a more potent, long‐lasting vaccine.^16^

In the meantime, social distancing has been implemented in many locales and by most accounts has been at least moderately successful in reducing the spread of COVID‐19.^17^ New and more high‐throughput tests have also been developed, both for determining the presence of an active infection via viral RNA and previous infection via antibody titer analysis.^18^, ^19^ Convalescent plasma therapy may also help improve outcomes in patients with severe COVID‐19, though availability may be limited due to the (albeit decreasing) scarcity of donor plasma and difficulty obtaining it.^20^, ^21^ There are also a number of postinfection therapeutics being evaluated in the clinic for their potential ability to reduce the severity of COVID‐19.^22^, ^23^ Most of these drugs are repurposed small molecule antivirals and immune‐modulating antibodies either already approved for other indications (e.g., chloroquine, hydroxychloroquine, ribavirin, favipiravir) while others have progressed through early stage clinical trials, but have not yet received FDA approval (e.g., remdesivir, galidesivir, leronlimab). While there are numerous reports of their in vitro efficacy, their therapeutic value for humans remains unclear at present. With COVID‐19 spreading at an alarming rate and the FDA helping to facilitate safety and efficacy testing, some of these drug trials should achieve sufficient enrollment to draw conclusions about their efficacy with appropriate statistical power.

If proven effective, these drugs offer a couple of key advantages from a rapid response perspective. First, there is vastly more safety data available for these drugs than for novel vaccines. These drugs have been used in hundreds to thousands of people for those that have entered Phase III trials to billions of people for marketed drugs with a long history of use.^24^ The number of patients enrolled in the Phase I Moderna/NIAID vaccine trial (45)^25^ pales in comparison, as would be expected at this stage. Second, the ability to be effective after exposure to SARS‐CoV‐2 enables clinical trials to dramatically narrow down the patient population to be treated and allows outcomes to be measured on the order of weeks. Contrast this approach with standard Phase III vaccination testing which requires a large cohort and long‐term follow‐up studies to confirm safety and efficacy.^26^ Lastly, they often have more broad spectrum activity, which makes it more likely that they will remain functional even if SARS‐CoV‐2 mutates rapidly, though that does not appear to be the case at present.^27^ Depending on their activity, these could even serve as tools to combat the next viral Disease X that arrives sometime in the future.^23^

Thus far, there has been little discussion about using these drugs as prophylactics rather than postexposure treatments, which is presumably due to their potential side effects. For example, chloroquine, a drug approved to treat a variety of ailments including malaria, has a small therapeutics index (only two‐ or threefold higher the daily dose) resulting in potentially fatal acute cardiovascular toxicity.^28^ Even with as‐directed use, it is associated with high frequencies of nausea, diarrhea, vomiting, muscle weakness, vision loss after prolonged use, and a bevy of other symptoms. The antiviral mechanism of chloroquine is unclear and potentially multifactorial, though some evidence suggests that prophylactic use prevents some viruses from infecting cells by disruption endosomal function.^29^, ^30^ Whereas there is little motivation for taking chloroquine preemptively in its current state due to severe side effects and uncertain benefits for COVID‐19, its use could potentially provide a net benefit when there is an active infection. However, based on recent studies using chloroquine in patients with COVID‐19, including a double‐blind Phase 2 clinical study in Brazil which had to be halted due to safety issues, it does not appear promising that the current formulation is suitable for use.^31^, ^32^ Hydroxychloroquine showed a similar lack of efficacy in a U.S. trial.^33^

Fortunately, there is a strong precedence for the value of pre‐exposure prophylaxis (PrEP) when the side effects of antivirals are low in the form of HIV drugs, such as Truvada (emtricitabine/tenofovir disoproxil). Truvada inhibits reverse transcriptase to prevent HIV from creating DNA from its RNA, thereby preventing it from integrating into the host cell genome and replicating.^34^ Because this enzyme is not native or necessary for human cell function, inhibition with Truvada is not associated with highly pervasive or severe side effects, enabling its widespread used as both a prophylactic and a postexposure therapy.^35^ However, Truvada itself is unlikely to have efficacy against SARS‐CoV‐2 because it does not encode or use reverse transcriptase in its replication process.^23^ If an effective drug for treating COVID‐19 with infrequent and/or mild side effects is identified, we may be able to rapidly transition to evaluating its use for PrEP. However, if that drug does have side effects, how can we reduce its toxicity while maintaining efficacy against COVID‐19 to create a favorable value proposition for prophylactic use? We may be able to reduce the undesirable side effects of a drug through medicinal chemistry, controlled release, or targeted delivery. Using medicinal chemistry to alter a drug's therapeutic window or prolong its biological half‐life is a tried and true approach with many examples of success. However, this direct chemical modification would be limited to altering small molecule drugs and often involves a slow and empirical development process to develop a single drug substance, which likely cannot be completed and fully tested within the duration of an outbreak.^36^ Alternatively, drug delivery systems are unique in their ability to provide solutions for drugs that have promise, but are not sufficiently safe in a traditional formulation to administer to patients. This can be achieved by improving absorption, increasing intracellular delivery, maintaining drug concentrations within a small therapeutic window, or providing a high drug gradient between the organ of interest (e.g., lungs) and systemic circulation. Though the potential impact of these strategies on the more than 100 drugs being evaluated for COVID‐19 is difficult to summarize concisely, Table 1 provides a general perspective on the properties of drugs that may benefit the most from targeted delivery or controlled release formulations.

**TABLE 1:** COVID‐19 drug categories and their potential for synergy with drug delivery systems

	Viral target	Indirect (host target)
Small molecules	• These drugs may have varying level of specificity and activity for viruses depending on their mechanism of action and how conserved the drug target is between viruses. • Highly suitable for rapid repurposing against novel viral pathogens, but new drug development unlikely on a timeline relevant for outbreak response. • Targeted delivery may not be useful for drugs with activity against a target that is unique to viral entry or replication; however, drugs with less specific activity could benefit from targeted delivery to limit side effects. • Controlled release devices would be easy to formulate because of the inherently stability of small molecules and may be especially useful for drugs with short half‐lives, small therapeutic indices, or expensive/complicated production processes.	• Often used for immune regulation. • Potentially broad activity for use in response to or to prevent many viral infections because they act on common host machinery. • Because they act on host cellular machinery, they often interfere with normal physiological function, sometimes resulting in undesirable off‐target effects. • Targeted delivery would enhance the local drug concentration at the site of infection (e.g., lungs) while maintaining a low systemic concentration, thus limiting side effects. • Controlled release devices would be easy to formulate because of the inherently stability of small molecules and may be especially useful for drugs with short half‐lives, small therapeutic indices, or expensive/complicated production processes.
Antibodies and other proteins	• Good candidates for repurposing against novel pathogenic viruses if they target conserved proteins (e.g., the coronavirus spike protein), but likely difficult to isolate, validate, and produce on the timeline of a viral outbreak. • Potentially more specific than small molecule drugs, leading to reduced off‐target effects. • Highly specific viral‐targeted proteins are unlikely to benefit a great deal from targeted or controlled release systems owing to potentially large therapeutic indices; however, less specific proteins may benefit from targeted delivery to avoid high concentrations in off‐target tissues • Controlled release devices may be difficult to develop because of the generally poor stability of proteins at 37°C for extended periods of time and may not be necessary for antibodies with long half‐lives, like endogenous IgG.	• Often used for immune regulation. • May be possible to determine safety prior to the outbreak of a novel pathogenic virus and thereby accelerate the timeline to implementation, though virus‐specific efficacy would of course need to be evaluated. • Antibodies that competitively bind with proteins on the patient's cells to prevent viral entry may disrupt their normal physiological function and therefore have undesirable effects. • Local delivery could help to limit abnormal physiological function to only the target tissue where it is having a beneficial antiviral effect. • Controlled release devices may be difficult to develop because of the generally poor stability of proteins at 37°C for extended periods of time and may not be necessary for antibodies with long half‐lives, like endogenous IgG.
siRNA and mRNA	• Can be rapidly customized for novel viral pathogens once the sequence is known and achieve somewhat predictable efficacy, though safety requires evaluation on a case‐by‐case basis. • Would benefit greatly from improved non‐viral delivery formulations since poor delivery efficacy would allow viruses to enter or replicate in cells that have not received RNA. • Given the similar nature of most siRNAs, and to a lesser extent mRNAs, formulations would likely be broadly applicable to future customized therapies. • Controlled release formulations may be challenging to develop due to the lack of RNA stability; however, if stability concerns can be overcome, prolonged release could help to maintain optimally altered expression. • The pulmonary delivery of mRNA encoding antibodies against a virus is being evaluated, though it is not clear that this would be meaningfully more effective than untargeted delivery since antibodies are secreted and circulate systemically.	• siRNA against cell surface proteins known to facilitate viral entry can be evaluated ahead of time to determine safety and suggest efficacy against related viruses to speed implementation against novel pathogenic viruses. • siRNA can be rapidly customized in response to identification of the host protein being used for cell entry or viral replication. • mRNA may be used to increase the expression of protective proteins. • In either case, efficient local delivery would be desired to avoid substantial modification of the patient physiology (e.g., systemic side effects) while maintaining efficacy at the site of viral replication and delivery. • Controlled release would be especially beneficial for prophylactic use if RNA stability concerns can be overcome through modification or other means.

The development of sustained release platforms could enable the use of an array of drugs that otherwise exhibit harmful side effects. For example, lopinavir and ritonavir, an HIV drug combination which is currently under evaluation as a COVID‐19 treatment, has common side effects that include diarrhea, nausea, and liver damage.^37^ These drugs have a half‐life of ~4–6 hr,^38^ meaning that systemic concentrations can vary by a factor of eight between peak and trough. Developing a controlled‐release formulation that exhibits zero‐order release kinetics to maintain the minimum effective drug concentration could mitigate these side effects by reducing the steady‐state drug concentration by as much as eight‐fold and reducing the hepatic processing burden by 81%. Although the ability to achieve zero‐order in vivo release kinetics with an oral or injectable delivery system largely remains elusive, even formulations that exhibit readily achievable first‐order release kinetics could assist in reducing toxicity. Not all drugs under evaluation for COVID‐19 are likely to benefit from this approach, however. Chloroquine, for example, has a biological half‐life of up to 50 days and thus peak‐to‐trough systemic drug concentrations are unlikely to vary dramatically between daily doses.^39^

Targeted drug delivery may offer a similar or even superior ability to reduce toxicity in some cases, particularly for respiratory infections. Because the lungs comprise only about 2% of total body weight, targeted delivery could decrease the amount of drug required by a factor of 50 or more compared with traditional oral administration once first‐pass metabolism is accounted for. One particularly promising approach is the hitchhiking of drug‐loaded nanocarriers on red blood cells.^40^ Intravenous administration of these constructs improved delivery to the lungs by ~40‐fold and therefore could be used to achieve an effective local concentration without requiring a high systemic drug concentration. The preparation of inhalable particles for local delivery is perhaps an even simpler approach, so long as the safety and utility concerns can be addressed.^41^ These strategies could provide safe and effective dosing even when there would otherwise be no therapeutic index (i.e., adverse events begin to occur before the drug is effective).^42^

An ideal drug formulation would exhibit high potency against SARS‐CoV‐2, have an excellent safety profile, and be produced via an inexpensive and scalable process. In addition, it would be very helpful if delivery systems were modular to enable their facile customization with new drugs. This could also enable a multidrug treatment to prevent the induction of resistance, which has been observed for some antivirals.^43^, ^44^ The co‐delivery of multiple drugs with different mechanisms of action simultaneously using either a combined (e.g., in the same particle) or preferably modular approach (e.g., blending particles containing different drugs) to enable novel virus flexibility could prevent viruses from developing resistance, including cross‐resistance.^45^Controlled‐release systems may be employed to ensure a consistent, effective level of drug is present to avoid applying a selective pressure for drug resistance without concerns over poor patient compliance.^46^ Similarly, targeted drug delivery systems could avoid dose‐limiting toxicity to ensure the effect of antivirals is sufficiently high to prevent the replication of all viral mutants present.^47^ Traditional controlled release and targeted delivery approaches may not be well‐suited for the delivery of biomacromolecular therapeutics due to their potential loss of higher order structure and thus bioactivity during formulation and release.^48^, ^49^ Fortunately, as of 2018, 77 of the 88 FDA‐approved antivirals were small molecules,^50^ which historically have been easier to formulate.^51^ In the best‐case scenario, we would have a formulation that acts on both SARS‐CoV‐2 as part of a broad spectrum of activity to have a therapy at the ready (i.e., tested for safety) for future outbreaks of novel viral pathogens, so that their efficacy against these pathogens could be rapidly evaluated and implemented to prevent or treat the disease.

After recent outbreaks including Ebola virus, Zika virus, severe acute respiratory syndrome‐related coronavirus (SARS‐CoV), middle east respiratory syndrome‐related coronavirus (MERS‐CoV), norovirus, N1N1pdm09 virus (swine flu), and a variety of avian flu viruses there was a flurry of activity to not only develop a vaccine, but also pre‐ and postexposure therapeutics. Unfortunately, or perhaps fortunately because outbreaks were mostly limited in duration and spread, these development efforts were largely unable to help with the outbreak that prompted their development. A nanoparticle formulation of ivermectin (a drug currently being explored for SARS‐CoV‐2 activity) that enhances intestinal absorption and exhibits controlled release to extend the duration of therapeutic drug levels was published 3 years after the end of the Zika virus outbreak for which it was intended.^52^, ^53^ Another series of papers showed the ability to limit the effects of Ebola virus after exposure using lipid nanoparticles to deliver siRNA targeting an Ebola virus protein.^54^, ^55^, ^56^ In the last of these papers, nonhuman primates still exhibited signs of advanced Ebola virus disease, but 100% survived whereas no animals in the control group survived. This work was published in April 2015, 14 months before that outbreak had ended, though the Ebola virus was well‐known before that 2‐year outbreak.^56^

However, this historical precedence for advanced formulations lagging behind the outbreak that stimulates their development may not hold true for COVID‐19 since there is no precedence for the magnitude of COVID‐19 in recent times or the resources being made available for its elimination.^57^, ^58^ On the spectrum of rapid response readiness, the repurposing of existing drugs with broad‐spectrum activity and known side effects that can be mitigated with advanced drug delivery techniques should be a top priority. Virus‐targeting small molecule antivirals may be easy enough to formulate and can be tested for efficacy against SARS‐CoV‐2 in parallel. However, interferon therapy, which targets the host immune system to reduce disease severity and has shown efficacy against SARS‐CoV‐2 in vitro,^59^ may pose a greater formulation challenge. In addition to protein stability concerns, the short biological half‐life and off‐target effects of interferons can yield severe and undesirable side effects when administered via traditional formulations.^60^, ^61^, ^62^ To overcome these obstacles, there has been a concerted effort to develop advanced interferon formulations ranging from sequestration in nanogels for oral delivery^63^ to implantable devices releasing interferons with zero order.^64^ Inhalation of atomized interferon alpha has been recommended by Chinese guidelines in some patients with COVID‐19 with uncertain results.^23^, ^65^

Beyond these “off‐the‐shelf” approaches, the next tier of priorities would be to employ platforms that can be easily customized to SARS‐CoV‐2, such as molecular imprinted polymers (MIPs) and nucleic acid therapeutics. MIPs, also referred to as synthetic antibodies, could be a direct substitute for convalescent plasma therapy.^66^ However, unlike convalescent plasma therapy, which is limited by the need for healthy, willing donors who have previously contracted the disease,^67^ MIP only requires a viral template, which can be generated created in a laboratory setting. This could be an especially important treatment in the early weeks of an outbreak when there is yet to be a sizable population of recovered patients. Nucleic acid therapies are particularly intriguing because of our ability to sequence a pathogenic viral genome soon after the outbreak has started and rapidly and inexpensively synthesize short RNA sequences as well as their potential to exhibit high specificity and be used after exposure. We have seen the inherent speed advantages of working with nucleic acids instead of proteins in the rapid production of a vaccine by Moderna and the NIAID, yet there is a long tail to those studies before efficacy can be determined. As a postexposure drug treatment, the efficacy of siRNA therapy could be evaluated in weeks rather than years. Whereas developing potent small molecule and protein therapeutics de novo in response to a viral epidemic (with or without advanced delivery platforms) does not appear possible on a relevant timeline, this generalizable approach seems much more well‐suited for rapid therapeutic development.

If we are able to develop these high‐efficacy, low‐toxicity formulations, the next question is, of course, who should be taking these drugs prophylactically and when should they take them? The answer likely depends on the residual side effects and severity of the disease they are preventing, though from an ethical standpoint it is pretty clear that any use should be voluntary. If they have an exceptional safety profile and it is cost‐effective to produce them, their use could be very widespread during periods of viral outbreak. If they are expensive, but effective or have a less clear net benefit to the average person, their distribution could be more targeted to high‐risk populations. Providing effective, low‐toxicity prophylactics to healthcare workers might be the most direct benefit to society. The value of healthcare workers in the face of a pandemic is well‐appreciated by most, but we must do a better job of providing them with safe working conditions than we have during the current COVID‐19 pandemic. These workers disproportionately interact with infected individuals, which increases their chance of contracting the disease. They also interact closely (and physically) with many people, which both increases their risk of contracting the disease and spreading it to others. Further, their frequent interaction with other healthcare workers creates the potential for a transmission nexus. Lastly, they also disproportionately interact with individuals likely to experience the worst COVID‐19 outcomes, such as immunocompromised patients and patients with other comorbidities.^68^ If we can augment the protection provided by PPE using pharmaceutical interventions, we may be able to stymie the spread of the disease and maintain a healthcare workforce operating at full capacity when they are most needed.

Even though deaths and infections appear to be approaching their apex in some areas thanks to increased awareness and social distancing, we are likely still in the early stages of life with COVID‐19. The worst wave of infections has still yet to hit many cities and countries, so it is too soon to estimate when we can resume normal societal operations, though some studies have painted a bleak outlook.^69^ With the work of tens of thousands of dedicated scientists, healthcare providers, and front line workers and some luck, our vaccine development efforts will pay dividends in short order and render the production of safer COVID‐19 treatments and prophylactics temporarily obsolete. However, if first‐generation vaccines prove ineffective or the SARS‐CoV‐2 virus mutates at a rate that prevents long‐lived immunity, drug formulations could help sooner than later. Regardless of the readiness of these formulations for the current COVID‐19 pandemic, we have now seen the havoc that a Disease X can wreak on our society and would be wise to develop both technology and social measures to mitigate the impact of the next Disease X. In some ways, we are fortunate that this virus is related to previous viral pathogens (MERS‐CoV and SARS‐CoV), which enabled us to have some basic understanding of this new virus as well as some tools ready in advance of its arrival.^23^, ^70^, ^71^ In other ways, such as SARS‐CoV‐2's propensity to remain asymptomatic, yet transmissible early in an infection,^72^ we were not. There are few certainties about what the next Disease X will look like; therefore, establishing broad‐spectrum pharmaceutical formulations to treat, or better yet prevent, infections may offer a key tool in future fights against novel viral pathogens.

## CONFLICT OF INTEREST

The author has no conflicts of interest to declare.
